# Researches on the Quantity of Carbonic Acid Exhaled from the Lungs of the Human Species

**Published:** 1843-07

**Authors:** MM. Andral


### RESEARCHES ON THE QUANTITY OF CARBONIC ACID EXHALED FROM THE LUNGS OF THE HUMAN SPECIES. By MM. ANDRAL and GAVARRET.

THIS paper gives the partial results of a very extensive series of experiments, on which the authors are engaged. If they continue as they have begun, there can be no doubt that their inquiries will tend greatly to the advancement of our knowledge of the respiratory function, in health and disease. Their first object has been to ascertain the modifying influence of *age, sex,* and constitution. To determine this, their observations have been made under circumstances as uniform as possible; the subjects of them having been in good health, and in similar condition as regards food, muscular expenditure, moral state, interval subsequently to the last meal, and hour of the day. Each experiment was repeated several times on the same subjects, and the accordance between the results has been as great as could be desired in physiological researches. The apparatus employed was so devised, as to enable the respirations to be freely performed; no portion of the expired air was again inspired; and the greatest care was taken to analyse the expired air with accuracy. The observations were continued, in each case, from about 8 to 13 minutes, until about 130 *litres* of gas were collected; and from these, the amount of carbonic acid thrown off per hour is calculated in terms of the quantity of solid carbon it contains. This is done, however, for the sake of comparison only, as it is not supposed by the authors that the means are thus afforded of estimating the whole quantity thrown off in 24 hours,—a question of which they reserve the consideration for the present. The following are the general results already obtained by them.

1. The quantity of carbonic acid exhaled by the lungs in a given time, varies according to the age, sex, and constitution of the subjects.

2. In both man and woman, the quantity undergoes modification according to the ages of the subjects experimented on, quite independently of their weights.

3. In all periods of life, comprised between 8 years and the most advanced old age, man and woman are distinguished by the difference in the amount of carbonic acid exhaled from their lungs in a given time. All other things being equal, man exhales a much larger quantity than woman. This difference is particularly well marked between the ages of 16 and 40 years, during which period the quantity of carbonic acid exhaled is nearly twice as much in man as in woman.

4. In man, the quantity of carbonic acid exhaled, continues to increase regularly from 8 to 30 years of age, and a remarkable increase takes place at the period of puberty. After 30 years, the exhalation of carbonic acid begins to decrease; and this decrease continues, becoming more and more marked, as the individual approaches nearer to extreme old age; so that, at this period, it returns to the standard at which it was about the age of 10 years.

5. In woman, the exhalation of carbonic acid augments according to the same laws as in man, up to the period of puberty; but at that epoch, the increase suddenly ceases; and the amount continues at this low standard, with little variation, as long as the catamenia make their regular appearance. But as soon as they cease, the exhalation of carbonic acid by the lungs undergoes a considerable increase; after which it decreases, as in man, in proportion as the age advances.

6. During the period of gestation, the amount of carbonic acid exhaled is temporarily raised to the standard which it attains after the cessation of the catamenia.

7. In both sexes, and at all ages, the quantity of carbonic acid exhaled by the lungs, is greater in proportion to the strength of the constitution, and the development of the muscular system.

The numerical results contained in the memoir itself have been collected by us in the following table, which expresses the quantity of solid carbon calculated to be exhaled in one hour. The gramme is equal to about 15½ grains.

| Male. | | Female. | | |
|---|---|---|---|---|
| 8 years | 5 grammes. | 8 years | 5 grammes. | The same standard continues in women during the whole of the menstrual period; but if the catamenia be temporarily suppressed, or pregnancy occur, it rises to the standard it attains after their entire cessation, namely 8·4 grammes. |
| 15 | 8·7 | 12-38 | 6·4 | |
| 16 | 10·8 | 38-50 | 8·4 | |
| 18-20 | 11·4 | 50-60 | 7·3 | |
| 20-30 | 12·2 | 60-80 | 6·8 | |
| 30-40 | 12·2 | 82 | 6·0 | |
| 40-60 | 10·1 | | | |
| 60-80 | 9·2 | | | |
| 102 | 5·9 | | | |

These numbers express the *averages;* the maximum amount is often considerably greater. In a young man of athletic system, and sound constitution, the quantity of carbonic acid exhaled in an hour was 14·1 grammes; a man of 60, equally vigorous for his age, threw off 13·6 grammes; and a man of 63 years,

12·4 grammes. An old man of 92 years, who preserved a remarkable degree of energy, and who had possessed an uncommon degree of vigour in his youth, was found to throw off 8·8 grammes per hour; whilst the same amount appeared to be the usual standard in a man of 45 years of age, who, unlike the preceding, had a feeble system, though in equally good health. The question how far these variations are connected with differences in the capacity of the chest, and in the number of respiratory movements, will be discussed in a future memoir.

*Annales des Sciences Naturelles.* Fevrier, 1843.

